# Greater preclinical atherosclerosis in treated monogenic familial hypercholesterolemia *vs*. polygenic hypercholesterolemia

**DOI:** 10.1016/j.atherosclerosis.2017.05.015

**Published:** 2017-08

**Authors:** Mahtab Sharifi, Elizabeth Higginson, Sven Bos, Angela Gallivan, Darren Harvey, Ka Wah Li, Amali Abeysekera, Angela Haddon, Helen Ashby, Kate E. Shipman, Jackie A. Cooper, Marta Futema, Jeanine E. Roeters van Lennep, Eric J.G. Sijbrands, Mourad Labib, Devaki Nair, Steve E. Humphries

**Affiliations:** a Centre for Cardiovascular Genetics, University College London, The Rayne Institute, University Street, London, WC1E 6JF, UK; b Department of Clinical Biochemistry, The Royal Free London NHS Foundation Trust, Pond Street, London, NW3 2QG, UK; c Department of Clinical Biochemistry, Russells Hall Hospital, The Dudley Group NHS Foundation Trust, West Midlands, DY1 2HQ, UK; d Department of Internal Medicine, Rotterdam, The Netherlands; e Inherited Cardiovascular Diseases, Institute for Cardiovascular Science, University College London, Paul O'Gorman Building, 72 Huntley Street, London, WC1E 6DD, UK

**Keywords:** Familial hypercholesterolemia, Polygenic hypercholesterolemia, Carotid intima media thickness, Coronary artery calcification, Preclinical atherosclerosis

## Abstract

**Background and aims:**

Familial hypercholesterolemia (FH) is a common inherited disorder of low density lipoprotein-cholesterol (LDL-C) metabolism. It is associated with higher risk of premature coronary heart disease. Around 60% of patients with a clinical diagnosis of FH do not have a detectable mutation in the genes causing FH and are most likely to have a polygenic cause for their raised LDL-C. We assessed the degree of preclinical atherosclerosis in treated patients with monogenic FH *versus* polygenic hypercholesterolemia.

**Methods:**

FH mutation testing and genotypes of six LDL-C-associated single nucleotide polymorphisms (SNPs) were determined using routine methods. Those with a detected mutation (monogenic) and mutation-negative patients with LDL-C SNP score in the top two quartiles (polygenic) were recruited. Carotid intima media thickness (IMT) was measured by B-mode ultrasound and the coronary artery calcium (CAC) score was performed in three lipid clinics in the UK and the Netherlands.

**Results:**

86 patients (56 monogenic FH, 30 polygenic) with carotid IMT measurement, and 166 patients (124 monogenic, 42 polygenic) with CAC score measurement were examined. After adjustment for age and gender, the mean of all the carotid IMT measurements and CAC scores were significantly greater in the monogenic than the polygenic patients [carotid IMT mean (95% CI): 0.74 mm (0.7–0.79) *vs.* 0.66 mm (0.61–0.72), *p* = 0.038 and CAC score mean (95%): 24.5 (14.4–41.8) *vs.* 2.65 (0.94–7.44), *p* = 0.0004].

**Conclusions:**

In patients with a diagnosis of FH, those with a monogenic cause have a higher severity of carotid and coronary preclinical atherosclerosis than those with a polygenic aetiology.

## Highlights

• Patients with clinical FH but no detectable mutation are likely to have a polygenic cause for their raised LDL-C.
• Preclinical atherosclerosis was measured in both groups, matched for lipid levels.
• Carotid Intima Media Thickness was greater in monogenic vs polygenic patients.
• Coronary artery calcification score was greater in monogenic vs polygenic patients.
• Monogenic FH patients have greater preclinical atherosclerosis than those with a polygenic aetiology.

## 1 Introduction

Familial hypercholesterolemia (FH) is a common autosomal dominant disorder and a well-known cause of premature coronary heart disease (CHD) [1]. It has a frequency of 1 in 200–500 in most European countries [2], [3], and is caused by mutations in the low density lipoprotein receptor (*LDLR*) gene, the gene coding for apolipoprotein B (*APOB*) or the gene encoding protein convertase subtilisin/kexin 9 (*PCSK9*) [4]. The clinical diagnosis of definite FH is based on a low density lipoprotein-cholesterol (LDL-C) level >4.9 mmol/L and the presence of tendon xanthomata, while patients with a diagnosis of possible FH do not have xanthomata but have a family history of premature CHD and/or hypercholesterolemia [5].

Only in 60–80% of definitive FH and in 20–30% of possible FH cases, can a mutation be found. [6], and since possible FH cases usually represent around two-thirds of the lipid clinic patient group, this means that overall a mutation can be found in roughly only 40% of those with a clinical diagnosis of FH [6]. It has been shown that a significant proportion of the patients with a clinical diagnosis of FH, where no mutation is found, are likely to have a polygenic explanation for their raised LDL-C level [7], [8]. A Global Genetic Consortium meta-analysis identified multiple loci where common variants associated with slight deviation in LDL-C levels [9]. Based on the common LDL-C raising single nucleotide polymorphisms (SNPs), a weighted SNPs score has been developed [7], [8]. Using this score, it appears that at least 20% of FH patients without a mutation are likely to have a polygenic explanation for their LDL-C level of over 4.9 mmol/L. In contrast, in individuals with a low LDL-C SNP score, there is a possibility that there may be a yet unidentified monogenic cause.

The elevated risk for CHD in FH patients with a detected mutation has been convincingly confirmed by Khera et al. in a population-based analysis, which showed that patients with LDL-C >4.9 mmol/L and no FH mutation had a 6-fold higher risk for CHD and those with both LDL-C >4.9 mmol/L and an FH mutation had a 22-fold higher risk compared to subjects with normal LDL-C and no mutation [10]. The Simon Broome register showed that the Standardized Mortality Ratio (SMR) for CHD in patients with a clinical diagnosis of definite FH was higher than in patients with a possible diagnosis of FH [SMR = 2.94 (2.28–3.80) *vs.* 2.05 (1.45–2.82)] [11]. Since we now know that a mutation can be found in 60–80% of definite FH patients, this means that the majority of this group had a monogenic cause, while the detection rate in possible FH is only 20–30% and they were likely to have polygenic hypercholesterolemia. Humphries et al. also reported a significantly higher Odds Ratio (OR) for having CHD in FH patients with an *LDLR* mutation *versus* patients where no mutation was found [OR = 1.84 *vs.* 1.00, *p* = 0.02] [12]. Several case control studies also reported the raised CHD risk in monogenic FH patients compared to the patients with a high LDL-C with no mutation found and the general population by means of the imaging measurements such as angiography, CT scan and carotid ultrasounds [13], [14], [15].

The European guideline for cardiovascular risk stratification recommends imaging techniques for intermediate and high risk asymptomatic individuals such as patients with FH [16]. Coronary artery calcium (CAC) score has long been recognized as a surrogate marker for coronary atherosclerosis and a good predictor of future cardiovascular events and all-cause mortality in asymptomatic people [17], [18]. Several clinical trials have shown that carotid IMT changes are sensitive to changes in the LDL-C levels [19]. A raised carotid IMT measurement is associated with increased risk of CHD and serves as an atherosclerotic surrogate end-point for therapeutic interventions [20].

While raised LDL-C is a known risk factor for atherogenesis [21], there are only limited data available to examine whether the extent of early atherosclerosis is higher in treated monogenic FH than in clinically diagnosed FH patients with the same level of LDL-C level but a polygenic cause. In this study, for the first time, we used the genetic testing to confirm the presence of the polygenic locus in individuals with a raised LDL-C, where no FH causing mutation was found and we compared the degree of preclinical atherosclerosis in these patients with monogenic FH patients.

## 2 Materials and methods

### 2.1 Subjects

Data from two outpatient lipid clinics in the UK, the Royal Free Hospital in London and the Russells Hall Hospital in Dudley, and an outpatient lipid clinic in the Netherlands, the Erasmus Medical Centre in Rotterdam, were included in this study over the period 2014–2016.

The following clinical diagnostic criteria for FH were used: an LDL-C level above the 95th percentile for gender and age in combination with the presence of tendon xanthomas in the patient or in a first degree relative, or an LDL-C level above the 95th percentile for gender and age in a first degree relative, or a proven coronary artery disease in a first degree relative under the age of 60. No patients had proven CHD or had any symptoms suggestive of ischemic heart disease.

All patients with secondary causes of hypercholesterolemia such as renal disease, liver disease and thyroid disease were excluded from the study. All the patients with a CHD disease or any symptoms of ischemic heart disease, renal insufficiency (serum creatinine > 120 mmol/L), known contrast allergy or atrial fibrillation were excluded from the study.

All patients had a genotyping test to confirm their monogenic or polygenic cause (see below) and they all had a CT scan to measure coronary calcium score or a carotid ultrasound to measure carotid intima media thickness. All patients were clinically asymptomatic, meaning they had no cardiac symptoms or any history of CHD. The inclusion age for the study varied from 30 to 70 years to have a carotid ultrasound and 40 to 70 for CT scan. All patients gave written informed consent. The ethical approval was obtained from the relevant ethics committees (13/LLO/1334).

Data from all the monogenic patients and the patients with no mutation and a gene score in the top two quartiles of *LDL-C* gene scoring were included in the final analysis of this study. The data from the patients with no mutation in genotyping and a low gene score were excluded from the analysis. The patients at the Rotterdam and Russells Hall hospital in the UK only underwent a CT scan to measure the CAC score, while the patients at the Royal Free hospital had only carotid IMT measurement. From a total number of 312 patients (94 patients at the Russells Hall and 97 at the Royal Free hospital in the UK and 121 patients in the Netherlands), data from 166 patients with a CAC score and 86 patients with a carotid IMT measurement were included in the final analysis.

### 2.2 Molecular analysis

#### 2.2.1 FH genotyping

All participants had FH mutation testing for all 18 exons of the *LDLR* gene, a fragment of exon 26 of *APOB* to cover the area for the common mutation p.Arg3527Gln, and exon 7 of *PCSK9* to cover p.Asp374Tyr using direct sequencing analysis of PCR products [22], [23], [24]. Multiplex Ligation-dependent Probe Amplification to detect gross deletions and insertions in *LDLR,* according to the manufacturer's protocol on all samples (MRC-Holland, Amsterdam, the Netherlands), and *in silico* prediction of pathogenicity of identified variants were also performed [25].

#### 2.2.2 LDL-C gene score calculations for polygenic hypercholesterolemia

The patients with no mutation detected in their FH genotyping test were genotyped for six LDL-C-raising SNPs (*CELSR2* (rs629301), *APOB* (rs1367117), *ABCG8* (rs4299376), *LDLR* (rs6511720), and *APOE* (rs429358, and rs7412)) at the Cardiovascular Genetics Lab at UCL in the UK. KASPar™ PCR technique (Kbiosciences, UK Hoddesdon, Herts, UK) or TaqMan^®^ assays (Life Technologies, Carlsbad, California, US) and genotype calls for all assays was carried out using an automated system, the results of which were checked manually by study personnel using SNP viewer^®^ software (Supplementary Table 1) as previously described [7], [8]. Patients were grouped into quartiles of the gene score based on those reported by Futema et al. for a healthy UK population. It has been estimated using probability calculations that patients in the top three quartiles of the score have a greater than 98% probability of having a polygenic cause of their hypercholesterolemia [8]. Only data from patients with an LDL-C SNP score in the top 2 quartiles were included in this study.

### 2.3 Biochemical markers

The lipid levels were measured based on the standard Lab techniques [26]. The biochemistry tests were performed on the automated Roche cobas^®^ and Vitros^®^ Fusion 5.1 analyser (Ortho-Clinical Diagnostics, Rochester, NY, U.S.A.) at Royal Free Hospital and the Russells Hall Hospital, respectively.

### 2.4 Carotid intima media thickness (IMT) measurement

The carotid IMT was measured in B-mode by a Philips CX50 machine equipped with a 5–10 MHz linear array probe at the Royal Free Hospital in the UK. Measurements were done in the far wall of the common carotid artery (in the second centimeter proximal to the bifurcation), the bifurcation and the internal carotid artery on both right and left arteries. Three scan angles of lateral, posterior and anterior during diastole were used, and each segment was measured in at least four different frames. IMT analysis was performed by Philips QLAB^®^ software after completed examination. In the case of plaque presence, the IMT was measured away from the plaque.

### 2.5 CT scan with coronary artery calcification (CAC) score

CAC was measured using Symbia TruePoint T6 SPECT/CT scanner (Siemens Medical Solutions, Forchheim, Germany) and dual-source CT scanner (first 101 scans: Somatom definition, last 44 scans: Somatom Definition FLASH, Siemens Medical Solutions, Forchheim, Germany) [15] in the UK and in the Netherlands, respectively. The CAC score measurement was done using the same standard Agatston calcium scoring algorithm [27].

CT scans of the heart (from the carina to the apex of the heart) were acquired during one inspiratory breath-hold without the use of contrast medium. CAC was quantified using calcium scoring software (Syngo CaScore, Siemens) and measurements were performed using the standard Agatston calcium scoring algorithm [27], which has been validated in several large studies. It has been shown previously that in asymptomatic individuals with a CAC score <100, the prevalence of cardiac ischemia is generally very low (<10%) [28], [29] Therefore, in our study, the participants were divided into two groups for comparison with the calcium score above and below 100 Agatston units.

### 2.6 Statistics

Demographic and biochemical data are presented as mean with standard deviation (SD) or number (percent). The carotid IMT and CAC score data were not normally distributed, so log-transformed data were used to compare the groups after adjustment for age and gender (SPSS^®^ version 21), and they were transformed back to the original scale and presented as geometric means and 95% confidence intervals.

For carotid IMT, a linear regression model was used. For CAC score, a tobit model was used due to the high frequency of zero scores. Values were recoded to CACS+1 to allow a censored threshold of zero in the tobit model. In addition, CAC score was analysed as two groups using a cut-point of 100 and an ordinal regression mode. The proportional odds assumption of the ordinal logistic regression model was satisfied by all variables (Brant test: age *χ*2 = 3.1 *p* = 0.38, sex *χ*2 = 3.96 *p* = 0.27, study *χ*2 = 0.96 *p* = 0.81, mutation status *χ*2 = 0.47 *p* = 0.93).

Logistic regression was used to adjust for age and gender for this analysis. Mutation, centre and gender were included as dummy variables in each model. The characteristics between the patient groups were compared using unpaired *t*-tests for continuous variables and Chi-squared tests for categorical data.

Linear regression and tobit regression models were also fitted using age, mutation and an age*mutation interaction term to determine differences between the increase in carotid IMT and CAC score with age, in monogenic and polygenic groups, respectively. Results are presented as beta coefficient (B) and standard error (SE). Based on the carotid IMT data in Jarauta et al. [30], a sample of 50 in each monogenic and polygenic group would give 80% power at the 5% significance level to detect an 11% difference in carotid IMT.

## 3 Results

### 3.1 Patient characteristics

In total, data from 252 patients were included in the study (Table 1). 86 patients (56 monogenic and 30 polygenic) had a carotid IMT measurement and 166 patients (124 monogenic and 42 polygenic) had a CAC score measurement. There was no significant difference between monogenic and polygenic groups in pre-treatment cholesterol levels in the UK centres but the pre-treatment LDL-C in the monogenic group was significantly higher in the Netherlands centre. There was no significant difference in other conventional cardiovascular risk factors such as smoking, hypertension, diabetes or body mass index between groups.

**Table 1:** Characteristics of the subjects studied.

**Characteristics of patients with carotid IMT measured**				
		**Monogenic (N = 56)**	**Polygenic (N = 30)**	***p***
**Male**	N (%)	22 (40)	14 (47)	0.3
**Age (years)**	Mean (SD)	50 (14)	57 (12)	0.03
**Pre-treatment lipid levels**				
TC (mmol/L)	Mean (SD)	8.1 (1.5)	8.2 (1.0)	0.5
LDL-C (mmol/L)	Mean (SD)	5.8 (1.6)	5.9 (0.9)	0.8
HDL-C (mmol/L)	Mean (SD)	1.5 (0.4)	1.9 (1.1)	0.1
TG (mmol/L)	Mean (SD)	1.2 (0.5)	1.6 (0.7)	0.01
**Tendon xanthoma**	N (%)	29 (51.7)	10 (33.3)	0.10
**Family history of premature CHD**^a^	N (%)	30 (53.5)	20 (66.6)	0.24
**BMI (kg/m**^**2**^**)**	Mean (SD)	26.1 (4.6)	26.4 (4.4)	0.1
**Patients with hypertension**	N (%)	4 (7.1)	1 (3.3)	0.1
**Patients with diabetes**	N (%)	0 (0)	1 (3.3)	0.3
**Smoker**	N (%)	0(0)	0(0)	
**Post-treatment lipid levels**				
TC (mmol/L)	Mean (SD)	4.8 (0.8)	5.0 (0.9)	0.3
LDL-C (mmol/L)	Mean (SD)	2.9 (0.8)	2.8 (0.8)	0.8
HDL-C (mmol/L)	Mean (SD)	1.5 (0.3)	1.6 (0.4)	0.2
TG (mmol/L)	Mean (SD)	0.8 (0.2)	1.3 (0.6)	0.001
**Patients on lipid lowering medication**	N (%)	42 (75)	25 (85)	0.7
**Years treated with statin**	Mean (SD)	10	8	0.2
**Characteristics of patients with CAC score measured UK data**				
		**Monogenic (N** = **49)**	**Polygenic (N** = **30)**	***p***
**Male**	N (%)	22 (44.9)	12 (40)	0.67
**Age (years)**	Mean (SD)	43.6 (9.8)	59.6 (8.1)	0.001
**Pre-treatment lipid levels**				
TC (mmol/L)	Mean (SD)	8.6 (0.8)	8.8 (1.3)	0.76
LDL-C (mmol/L)	Mean (SD)	6.3 (0.7)	6.1 (1.1)	0.66
HDL-C (mmol/L)	Mean (SD)	1.5 (0.4)	1.7 (0.5)	0.3
TG (mmol/L)	Mean (SD)	1.8 (0.8)	2.3 (1.2)	0.32
**Tendon xanthoma**	N (%)	30 (61.2)	3(10)	<0.001
**Family history of premature CHD**^a^	N (%)	30 (61.2)	15 (50)	0.03
**BMI (kg/cm**^**2**^**)**	Mean (SD)	27.7 (4.4)	29.7 (9.2)	0.58
**Patients with hypertension**	N (%)	1 (14)	12 (40)	0.3
**Patients with diabetes**	N (%)	0 (0)	3 (10)	0.9
**Smoker**	N (%)	1 (14.3)	4 (13.0)	1.0
**Patients on lipid lowering medication**	N (%)	75 (100)	23 (76)	0.1
**Years treated with statin**	Mean (SD)	9.0 (7.5)	3.0 (3.0)	0.006
**Characteristics of patients with CAC score measured Rotterdam data**				
		**Monogenic (N** = **75)**	**Polygenic (N** = **12)**	***p***
**Male**	N (%)	52 (69.3)	8 (66.7)	0.85
**Age (years)**	Mean (SD)	51.4 (7.7)	55.8 (8.6)	0.07
**Pre-treatment lipid levels**				
TC (mmol/L)	Mean (SD)	9.92 (2.29)	8.72 (2.00)	0.09
LDL-c (mmol/L)	Mean (SD)	7.64 (2.12)	6.22 (1.76)	0.03
**Tendon xanthoma**	N (%)	28 (37.3)	0	–
**Family history of premature** **CHD**^a^	N (%)	36 (48)	7 (58.3)	0.50
**BMI (kg/cm**^**2**^**)**	Mean (SD)	26.7 (3.8)	24.6 (3.2)	0.07
**Patients with hypertension**	N (%)	15 (20.0)	5 (41.7)	0.1
**Patients with diabetes**	N (%)	2.7 (2)	8.3 (1)	0.36
**Smoker**	N (%)	14 (18.7)	3 (25.0)	0.7
**Post-treatment lipid levels**				
TC (mmol/L)	Mean (SD)	5.50 (1.48)	5.27 (1.79)	0.62
LDL-C (mmol/L)	Mean (SD)	3.55 (1.31)	2.89 (1.34)	0.11
HDL-C (mmol/L)	Mean (SD)	1.33 (0.39)	1.37 (0.38)	0.7
TG (mmol/L)	Mean (SD)	1.22 (0.87)	2.14 (3.58)	0.06
**Patients on lipid lowering medication**	N (%)	75 (100)	11 (91.7%)	0.14
**Years treated with statin**	Mean (SD)	10.8 (7.6)	5.8 (7.6)	0.04

a In 1st degree relative (<60 years old) or 2nd degree relatives (<50 years old).

### 3.2 Carotid intima media thickness (carotid IMT)

As shown in Table 2, the mean of all carotid IMT readings (mean IMT) was 12% higher in monogenic than polygenic patients after adjustment for age and gender [0.74 mm (0.7–0.79) *vs.* 0.66 mm (0.61–0.72), *p* = 0.038]. Similar differences were seen in the different segments analysed, with those of mean bifurcation IMT and mean internal carotid artery IMT being statistically significant.

**Table 2:** The mean and max carotid IMT in each carotid segment and the coronary artery calcium (CAC) score in monogenic and polygenic groups after adjustment for age and gender.

**Carotid IMT results**	**Monogenic** **(N = 56)**	**Polygenic** **(N = 30)**	***p***
	**mean (95% CI)**	**mean (95% CI)**	
Mean IMT^a^ (mm)	0.74 (0.70–0.79)	0.66 (0.61–0.72)	0.03
Mean CCA^b^ IMT (mm)	0.65 (0.61–0.68)	0.62 (0.58–0.66)	0.3
Max CCA^b^ IMT (mm)	0.72 (0.68–0.77)	0.70 (0.64–0.76)	0.5
Mean bifurcation IMT (mm)	0.81 (0.74–0.89)	0.70 (0.62–0.79)	0.05
Max bifurcation IMT (mm)	0.96 (0.85–1.07)	0.80 (0.69–0.93)	0.08
Mean ICA^c^ IMT (mm)	0.74 (0.66–0.83)	0.60 (0.52–0.7)	0.04
Max ICA^c^ IMT (mm)	0.82 (0.69–0.96)	0.65 (0.52–0.81)	0.1
N(%) patients with carotid plaque	12 (21%)	4 (13%)	0.4

**CAC score results** (N for monogenic/N for polygenic)	**Monogenic** **(N** = **124)**	**Polygenic** **(N** = **42)**	***p***
	**mean (95% CI)**	**mean (95% CI)**	
UK centre (49/30)	33.45 (13.9–81.5)	1.05 (0.32–3.44)	
Netherlands centre (75/12)	22.9 (12.1–43.4)	11.1 (2.3–54.0)	
Total	24.5 (14.4–41.8)	2.65 (0.94–7.44)	0.0004

CI, confidence interval.

a Mean IMT, mean of all carotid IMT readings.

b CCA, common carotid artery.

c ICA, internal carotid artery.

**Fig. 1:**
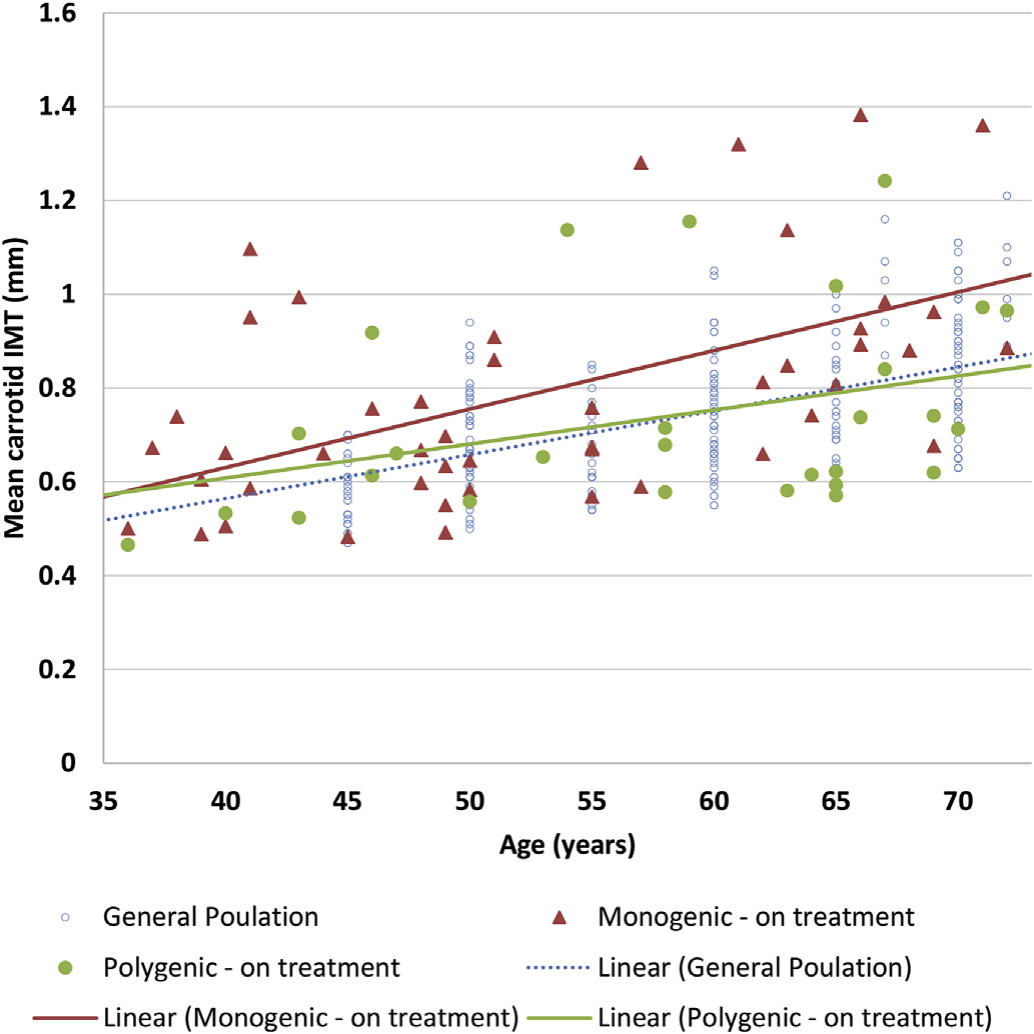
Mean carotid IMT measurements against age in treated monogenic FH, polygenic hypercholesterolemia, and the general population ^a^. ^a^ Scattergram of measures of mean carotid IMT in monogenic and polygenic subjects by the age at recruitment of the subjects. General population data obtained from Stein et al. The coefficient of determination (r^2^) between age and IMT values is the proportion of the variance in carotid IMT that can be explained by differences in age (r^2^ for mutation positive = 0.27, r^2^ for mutation negative = 0.08 and r^2^ for general population = 0.22). The value of 0.27 for mutation positive indicates that 27% of the variability in IMT can be explained by age variations, with 73% of the variability unexplained.

As expected, and as shown in Fig. 1, the carotid IMT significantly increased with age in both monogenic and polygenic groups compared to the general population. The increase was borderline significant (*p* = 0.057) over the age of 51 (median age) in the monogenic group. Comparing the carotid IMT in patients <51 *versus* ≥51 years old gave a mean (SD) carotid IMT of 0.63 mm (0.15) *vs.* 0.88 mm (0.24) in the monogenic group (*p* = 0.0005) and 0.60 mm(0.12) *vs.* 0.75 mm(0.19) in the polygenic group (*p* = 0.01).

### 3.3 Coronary artery calcium (CAC) score

Data for the CAC score is shown in Table 2. The CAC score did not differ, overall, between the UK *vs.* Netherlands centre (14.2 *vs.* 19.1, *p* = 0.50) and both centres showed a significantly higher CAC score in monogenic patients. The estimated mean (95% CI) CAC score in all centres was 24.5 (14.4–41.8) for the monogenic group, which was significantly higher than 2.65 (0.94–7.44) for the polygenic group (*p* = 0.0004) (Table 2). The CAC score was estimated to be 9.27 (95% CI: 2.74 to 31.4) times higher in the monogenic compared to the polygenic group after adjustment for centre, age and gender (*p* = 0.0004).

**Fig. 2:**
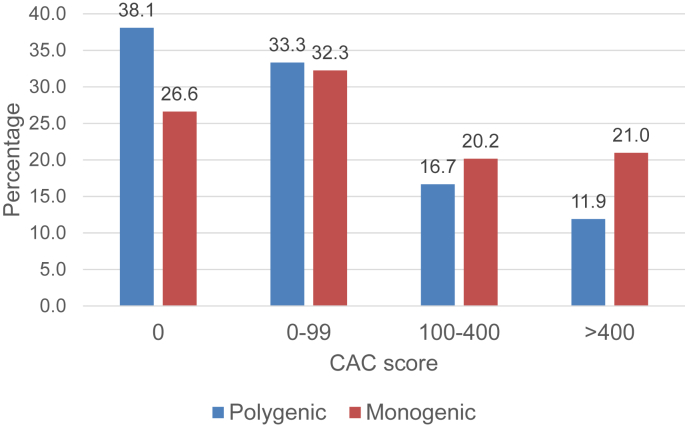
Percentage of monogenic and polygenic patients in each CAC score category ^a^. ^a^ The percentage of monogenic patients and polygenic patients within each CAC score category was estimated.

Fig. 2 shows the number of monogenic and polygenic patients in each CAC score category: zero, 1–99, 100–400 and > 400. A CAC score above 100 occurred in 51 (41.1%) monogenic patients compared to 12 (28.6%) polygenic patients with an odd ratio of 1.38 (*p* = 0.43). After adjustment for centre, age and gender, the odds ratio for having a CAC score >100 increased to 4.79 (95% CI: 1.67–13.75, *p* = 0.004). A CAC score of zero was reported in 33 (26.6%) monogenic patients compared with 16 (38.1%) polygenic patients (age, study, gender adjusted *p* = 0.01) and a CAC score >400 was found in 26 (21.0%) monogenic patients in comparison to 5 (12.2%) in the polygenic group (age, study, gender adjusted *p* = 0.03). CAC score data was also available for 49 excluded subjects with an LDL-C SNP score below our recruitment cut-off. As shown in Supplementary Table 2, *post-hoc* analysis suggests that those with no detectable mutation and a low LDL-SNP score have a CAC distribution that is more similar to the polygenic subjects than the monogenic patients.

## 4 Discussion

Our study showed that residual preclinical atherosclerosis, as measured in carotid and coronary arteries, was greater in treated asymptomatic monogenic FH patients compared to individuals with a polygenic aetiology.

Lipid-lowering therapy has showed an improved clinical outcome in patients with CHD both in primary and secondary prevention [31]. In our study, despite the longer lipid-lowering therapy for primary prevention in the monogenic group, both the carotid IMT and the CAC score remained significantly raised compared to the polygenic group. We also found a significantly higher number of monogenic patients with CAC score >400, while there was no significant difference in other cardiovascular risk factors among the groups. It would appear that the aetiology for raised LDL-C in these patients play a role in determining the degree of preclinical atherosclerosis. This could be explained by the substantially higher accumulated ‘LDL-C burden’ in monogenic patients since these patients have had genetically-determined lifelong high LDL-C [32]. In comparison, the LDL-C level in patients with polygenic hypercholesterolemia may only reach the LDL-C threshold of monogenic FH patients later in life after exposure to environmental factors (Humphries and Futema et al. unpublished).

The CAC score of zero has been reported in a range of 40–60% in the general population, in previous population-based studies [33], [34]. In our study, we found zero calcium score in 26.6% of patients with monogenic FH patients, which was significantly lower than the polygenic patients (38.1%) and the general population. Notably, the zero CAC score does not exclude the presence of atherosclerosis, but a previous study of FH patients showed the presence of non-calcified plaque only in 4% of FH patients with zero CAC score, and they all had less than 50% luminal obstruction [35].

Carotid IMT has been shown to be thicker in children with FH than in their unaffected siblings [36], and in adults with a known FH causing-mutation compared to the general population [37], [38], or patients with familial combined hypercholesterolemia [39]. Our results show that in treated monogenic patients, carotid IMT remains raised compared to treated polygenic patients and the general population throughout adulthood, although there was no significant difference in the percentage of patients with carotid plaque. This result should be viewed with caution since it is based on cross-sectional data, and validation of this using multiple measures is required. A total sample size of around 600 would be required to detect a significant (80% power at *p* = 0.05) interaction effect between age and carotid IMT thickness, comparing monogenic with polygenic subjects.

In all three groups, the monogenic subjects were younger and had been treated with statins for longer than the polygenic patients. While there is some evidence that statin treatment itself may be associated with high levels of coronary calcium (possibly as the plaque becomes lipid depleted and more stable) [40], we believe it is unlikely that differences in lipid-lowering treatment in the two groups explain the differences seen here.

The principle limitation of this study is the relatively small sample size. However, a strength of our study is that, in patients with a clinical diagnosis of FH, two different measures of atherosclerosis burden (carotid IMT and CAC score), in three completely independent centres, consistently found a lower burden in patients with a polygenic compared to a monogenic aetiology. Although all subjects fulfilled the clinical diagnosis of FH, it is possible that a small proportion of those designated as “polygenic” may carry an FH-causing mutation in the *LDLR/APOB/PCSK9* genes that has been missed because of technical reasons in the methods used for mutation detection, although this is unlikely to be more than 1–2 for each of the study groups. The inclusion of a few monogenic FH patients in the polygenic group would mean that the measured mean levels of carotid IMT or CAC score would be higher than that in a “pure” polygenic group, and as such could not be a confounder of the differences seen here. Conversely, since all the identified mutations in this group have been previously reported as FH-causing, there is likely to be no inclusion of any “false-positive” cases in the monogenic group.

Clearly, the differences in carotid IMT and coronary calcification seen here should be confirmed in a larger sample, and further studies of the coronary atherosclerosis burden would strengthen the inference. If it is confirmed that monogenic FH patients have higher residual atherosclerosis than polygenic ones, then it would be essential to have a better screening programme for FH patients. The availability of next generation sequencing for genetic screening and the non-invasive imaging techniques in clinical settings would help clinicians to prioritize the candidates with monogenic FH for better cardiovascular risk stratification and appropriate utilisation of funds in health services (e.g., use of novel therapeutic agents, such as PCSK9 monoclonal inhibitors).

## Conflict of interest

The study was supported by a grant from the British Heart Foundation (grant RC 93008). SEH and JAC acknowledge BHF support (RG 2008/008) and also funding from the Department of Health's NIHR Biomedical Research Centers funding scheme. MS was supported by the BHF and the Royal Free Hospital Charity. MF is funded by the ‘Fondation Leducq’ grant (14 CVD03). EJGS was supported by Dutch Heart Foundation (Grant 2006T102). DN has received grants from Pfizer (Pfizer Foundation award 2008), Solvay, Merck Sharp & Dohme and AstraZeneca. DN has advisory board membership for Merck Sharp & Dohme, Sanofi and Amgen.

## Author contributions

MS, EH, SB, AG, KES and DH were involved in recruitment and data collection. MS, KWL and MF performed the lab analysis and data interpretation. JAC did the statistical analysis. DN and SEH were involved in study design. MS wrote the manuscript and all authors were involved in critical revision of the paper.
