# Supplementary material for: Greater preclinical atherosclerosis in treated monogenic familial hypercholesterolemia vs. polygenic hypercholesterolemia
Source: Atherosclerosis. 2017 Aug;263:405–11. doi: 10.1016/j.atherosclerosis.2017.05.015 (PMC5567405; doi:10.1016/j.atherosclerosis.2017.05.015)
Supplement: Supplementary material [file mmc1.docx]

**Supplementary**

Table S1: Global Lipid Genetic Consortium six SNP LDL-C gene score calculations

|  | **Chromosome** | **Gene** | **Minor allele** | **Common allele** | **Weight for score calculation** |
| --- | --- | --- | --- | --- | --- |
| rs629301 | 1 | *CELSR2* | G | T ^a^ | 0.15 |
| rs1367117 | 2 | *APOB* | A ^a^ | G | 0.1 |
| rs4299376 | 2 | *ABCG8* | G ^a^ | T | 0.071 |
| rs6511720 | 19 | *LDLR* | T | G ^a^ | 0.18 |
| rs429358 | 19 | *APOE* | C | T | . |
| rs7412 | 19 | *APOE* | T | C | . |
| ε2ε2 | 19 | *APOE* | . | . | -0.9 |
| ε2ε3 | 19 | *APOE* | . | . | -0.4 |
| ε2ε4 | 19 | *APOE* | . | . | -0.2 |
| ε3ε3 | 19 | *APOE* | . | . | 0 |
| ε3ε4 | 19 | *APOE* | . | . | 0.1 |
| ε4ε4 | 19 | *APOE* | . | . | 0.2 |

^a^ Risk alleles (LDL-C raising)

Table S2: Distribution of those with different CAC scores in monogenic and polygenic patients with a low and high gene score

|  | Number of patients with different CAC score | | | |
| --- | --- | --- | --- | --- |
|  | 0 | 1-99 | 100-400 | >400 |
| No mutation detected, low gene score (n = 49) | 18 | 18 | 6 | 7 |
| Polygenic (n = 42) | 16 | 14 | 7 | 5 |
| Monogenic (n = 124) | 33 | 40 | 25 | 26 |

The Chi-squared analysis show that the distribution of CAC score >100 in the no mutation/low score (excluded) group is not significantly different from the polygenic group (*p*=0.8) and borderline different from the monogenic group (*p*=0.06).
